# Thoracic cavernous hemangioma mimicking an epidural neurinoma in an hourglass shape: a case report and review of the literature

**DOI:** 10.11604/pamj.2025.50.83.40589

**Published:** 2025-03-24

**Authors:** Dahmane Elhairech, Mohamed Lmejatti

**Affiliations:** 1 Neurosurgery Department, Souss Massa University Hospital, Agadir City, Morocco,; 2 Faculty of Medicine and Pharmacy of Agadir, Ibn Zohr University, CF49+F65, Agadir 80000, Morocco,; 3 Laboratory of Cellular Biology and Molecular Genetics, Faculty of Sciences, Ibn Zohr University, Agadir, Morocco

**Keywords:** Cavernous hemangioma, epidural neuroma, hourglass appearance, vascular malformation, case report

## Abstract

Epidural cavernous hemangioma is a rare vascular malformation, accounting for 5-12% of all spinal cord vascular malformations. We report a case of low dorsal spinal cord compression at D5-D6 due to a cavernous hemangioma mimicking the clinical and radiological features of an epidural neuroma in an hourglass shape. The clinical presentation was progressive spastic paraplegia over six months in a 41-year-old patient with no significant medical history. Magnetic resonance imaging showed a compressive D5-D6 intradural lesion with T2 hyperintensity, displaying an hourglass appearance suggestive of an epidural neuroma, with a significant extracanal component filling the ipsilateral latero-vertebral space. Histological examination confirmed cavernous hemangioma by immunohistochemistry. The postoperative outcome was favorable following total surgical removal of the lesion.

## Introduction

Cavernous hemangiomas, also known as cavernomas, are vascular malformations of the central nervous system, representing 5-12% of all spinal cord vascular malformations [1]. The association of both cerebral and spinal involvement is extremely rare [2]. The advent of magnetic resonance imaging (MRI) has significantly increased the number of diagnosed cases [3,4]. We present a case of histologically confirmed dorsal spinal cavernoma followed in our institution.

## Patient and observation

**Patient information:** we report the case of a 41-year-old female patient with no significant medical history. Six months before consultation, she developed fatigue in both lower limbs, followed by progressive gait disturbances, unstable walking, and a significant reduction in walking distance, with intermittent neurological claudication. Notably, there were no associated symptoms such as nocturnal back pain, fever, or weight loss.

**Clinical findings:** on admission, neurological examination revealed spastic paraparesis rated 2/5, with a sensory level up to D5, brisk and polykinetic osteotendinous reflexes, a bilateral Babinski sign, and abolition of abdominal skin reflexes. The deep sensation was absent in both lower limbs.

**Figure 1:**
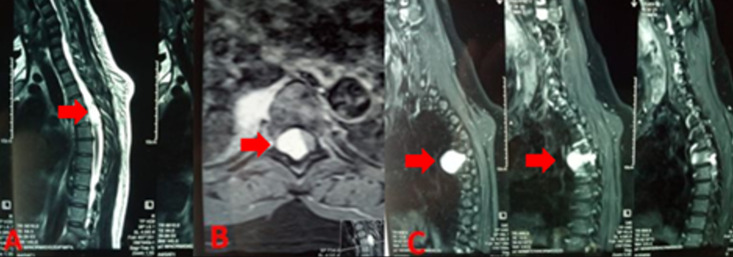
medullar MRI sequence T2 A) intradural right pseudocystic lesion at D5-D6, arrows indicate the lesion's location and effect on the spinal cord; B) same intradural lesion at D5-D6, arrows indicate the lesion and its relationship to the spinal cord; C) lesion extending through the D5-D6 foramen into the right lateral vertebral space, creating an hourglass appearance, arrows point to the lesion's outline and its extension into the lateral space

**Diagnostic assessment:** magnetic resonance imaging revealed a single intradural right pseudocystic lesion, oval with a large vertical axis, displacing and laminating the spinal cord on the left. The lesion was discretely hypointense on T1 and hyperintense on T2 compared to the spinal cord. After gadolinium injection, the lesion enhanced intensely and homogeneously, extending from D5-D6 foramina to fill the right latero-vertebral space, creating an hourglass appearance (Figure 1A, B, C).

**Figure 2:**
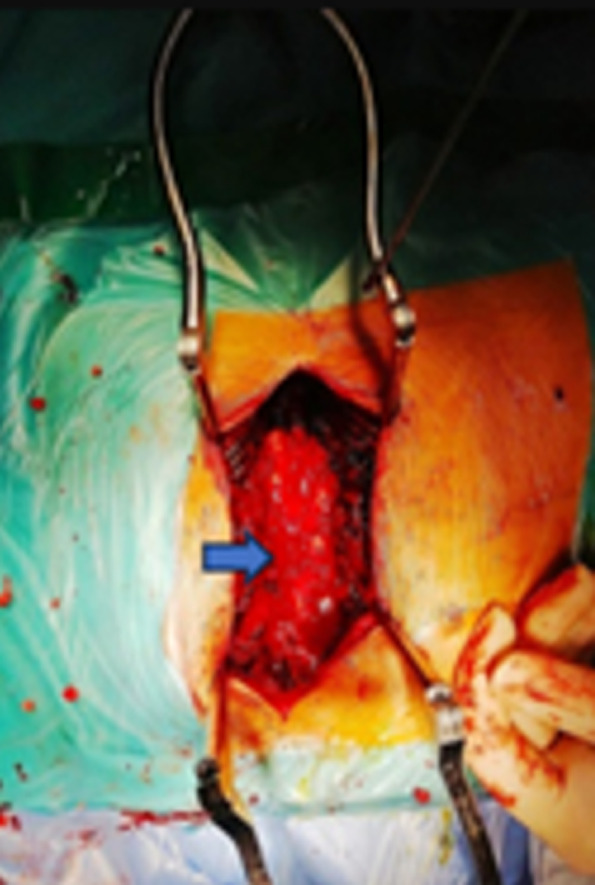
neurosurgical procedure intraoperative image showing the patient in the prone position during the neurosurgical procedure; a D5-D6 laminectomy was performed to remove a friable, bleeding mass near the right D5 root (arrows highlight the lesion and the surgical site)

**Therapeutic intervention:** given the clinical and radiological findings, a diagnosis of dorsal intradural hourglass neuroma was made. Surgical resection was performed in two stages. First, the patient was placed in the prone position, and a D5-D6 laminectomy was performed to remove a friable, bleeding mass involving the right D5 root. The second stage involved the patient being placed in the left lateral decubitus position, with selective intubation, followed by a posterolateral thoracotomy to remove the mass (Figure 2).

**Figure 3:**
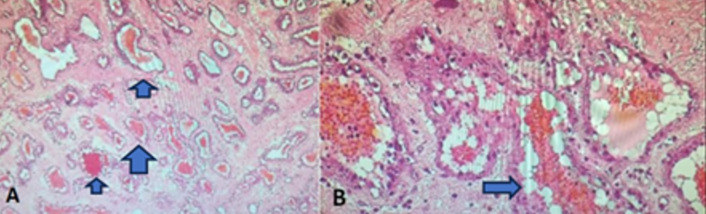
histological examination of the lesion: A) hematoxylin-eosin staining, magnification x4; arrows indicate the vessels and their characteristics; B) hematoxylin-eosin staining, magnification x20; vascular walls are lined by a single layer of turgid endothelial cells without cytological atypia, confirming the diagnosis of cavernous hemangioma (arrow highlights the endothelial lining)

**Follow-up and outcome:** histopathological examination of the tissue shows vascular proliferation with large, congested vessels and gaping lumens, characteristic of a cavernous hemangioma. Immunohistochemical staining for EMA and S100 was negative, ruling out a neurofibroma and confirming the diagnosis of cavernous hemangioma (Figure 3A, B). The postoperative course was uncomplicated, with rapid and complete recovery of the motor deficit and the patient's ability to walk independently.

**Patient’s consent:** informed consent was obtained from the patient and his family.

## Discussion

Cavernous hemangiomas of the central nervous system are characterized by capillary-type vascular malformations, with an overall prevalence of 0.5% in the general population, of which only 10% are familial. Intramedullary forms are rare and account for 5% of cerebral cavernomas [2]. The dorsal spinal cord is the most common location, accounting for 54% of cases, with 30% located in the high dorsal region and 24% in the low dorsal region [1]. Cervical lesions are less frequent (39%), while lumbar locations are rare (7%) [1]. Most patients present around the age of 40 [1,3,5,6-10].

In a retrospective multicenter study by Parker *et al*. 53 cases of intramedullary cavernoma were identified, with 38% presenting acutely and 60% progressively [10]. Triggering factors were reported in 26% of cases, including intense physical activity, pregnancy, and trauma. The clinical course of spinal cord lesions tends to be more aggressive than that of cerebral lesions, as the spinal cord has less tolerance for mass effects [3,4]. MRI is the most sensitive imaging technique, typically revealing an intradural lesion with a heterogeneous, reticulated signal center surrounded by a hypointense border due to hemosiderin deposition [2,6]. Treatment for symptomatic patients includes surgery, especially for superficial, exophytic, or posterior lesions. For deep lesions with minimal symptoms, conservative management with close monitoring is recommended. However, surgery is considered if there is evidence of lesion growth on follow-up imaging [7,8]. Complete resection is preferred to prevent recurrence and continued symptom progression. In a study by Parker *et al*. 54% of surgically treated patients showed durable improvement, and 87% of posterior intramedullary cavernomas had improved clinical outcomes after surgery [10].

## Conclusion

Cavernous hemangioma is a rare vascular malformation of the central nervous system, particularly in the spinal cord. MRI is crucial for radiological diagnosis. In our case, the cavernous hemangioma mimicked the appearance of an epidural neuroma in an hourglass shape. Total surgical removal is the treatment of choice and significantly improves functional prognosis.
